# Case Report: Application of Thoracoscopic Clamp Radiofrequency Ablation on Atrial Tachycardia Originating From Right Atrial Appendage After Catheter Ablation Failure

**DOI:** 10.3389/fcvm.2021.659821

**Published:** 2021-04-28

**Authors:** Li Luo, Zuoan Qin, Ruizheng Shi, Liangqing Ge

**Affiliations:** ^1^Department of Cardiology, Changde First People's Hospital, Changde, China; ^2^Department of Cardiology, Xiangya Hospital, Central South University, Changsha, China

**Keywords:** atrial tachycardia, tachycardia cardiomyopathy, radiofrequency ablation, right atrial appendage, catheter ablation

## Abstract

Atrial tachycardia originating from the right atrial appendage has a higher probability of failure of catheter ablation. Here we report a case of a 13-year-old boy with incessant tachycardia, complicated by heart enlargement, and heart failure. Electrophysiological examination showed that atrial tachycardia (AT) originated from the apex of the right atrial appendage, and endocardial catheter ablation was ineffective. After thoracoscopic approach, the right atrial appendage was successfully ablated with bipolar radiofrequency ablation forceps, atrial tachycardia was terminated and sinus rhythm was restored. Within 3 months since the patient was discharged from the hospital, no arrhythmia occurred and the heart structure returned to normal. Thus, thoracoscopic clamp radiofrequency ablation may be a reasonable choice for young patients with atrial tachycardia originated from the right atrial appendage when transendocardial ablation is not effective.

## Introduction

The right atrial appendage (RAA) is a rare source of atrial tachycardia. Although, there are a few cases where catheter ablation has successfully cured right atrial tachycardia (1, 2), most studies still believe that atrial tachycardia originating from the right atrial appendage, especially the apex of the right atrial appendage, has a higher probability of failure of catheter ablation (3, 4). Thoracoscopic clamp radiofrequency ablation is currently applied for pulmonary veins isolation to treat atrial fibrillation (5, 6), it can be combined with endocardial catheter ablation and improve the success rate of refractory atrial fibrillation ablation (7). Here we reported a 13-year-old boy with refractory atrial tachycardia originating from the apex of the right atrial appendage, complicated by cardiac dilatation, and heart failure. The drug treatment was ineffective. After endocardial radiofrequency catheter ablation failed, the patient underwent thoracoscopic clamp radiofrequency ablation.

## Case

**Figure 1:**
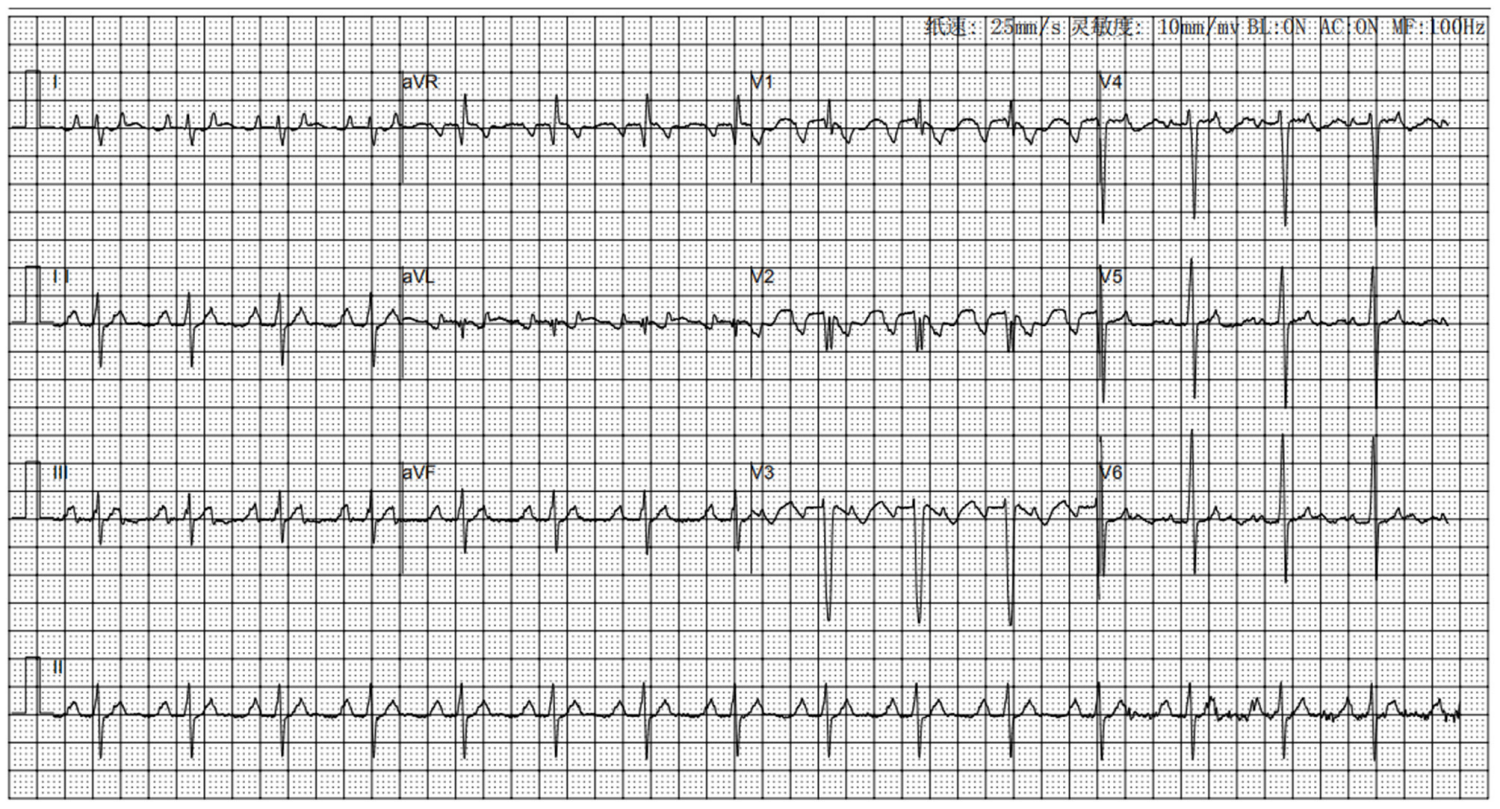
The surface electrocardiogram during the onset of atrial tachycardia.

A 13-year-old boy was admitted with palpitations and chest tightness for 2 years, which worsened for 10 days. The patient started to experience palpitations, fatigue, dyspnea, and chest tightness after repeated activities 2 years ago. The above symptoms have worsened in the past 10 days without any obvious cause. After admission, he was diagnosed with atrial tachycardia, cardiac dilatation, and heart failure. The value of NT-proBNP was 6,760 ng/L. Echocardiogram showed that the whole heart was enlarged. The diameter of left atrium and ventricle were 43 and 62 mm, respectively, and the left ventricular ejection fraction was 31%. The electrocardiogram showed atrial tachycardia. The P wave pattern was negative in lead V1, and the inferior lead was positive and towering, suggesting the origin of the right atrial appendage (Figure 1). The 24-h dynamic electrocardiogram showed persistent atrial tachycardia with variable conduction (2-1:1) and a maximum heart rate of 217 beats per minute. The use of digoxin and amiodarone to treat atrial tachycardia was not effective.

**Figure 2:**
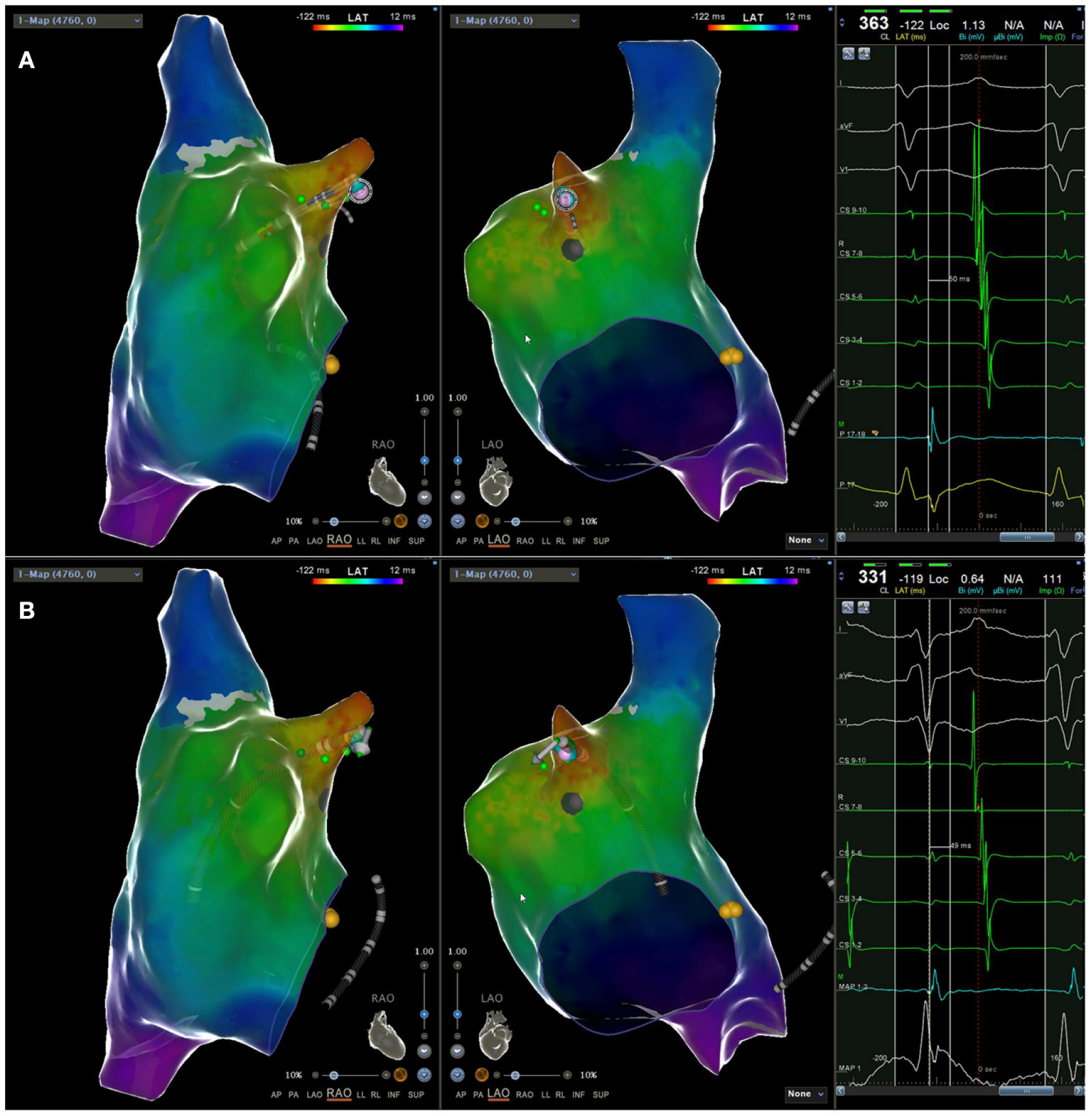
The earliest activation point (located at the apex of the right atrial appendage) measured by **(A)** the Pentaray electrode 50ms ahead of the body surface P wave; **(B)** the ST ablation catheter 49 ms ahead of the body surface P wave.

After admission, the patient underwent electrophysiological examination and radiofrequency catheter ablation. The standard catheter position was used for electrophysiological examination in the coronary sinus and His bundle area. Coronary sinus mapping showed that the sequence of atrial activation during atrial tachycardia was from proximal to distal, which was considered to be originated from the right atrium. Under the guidance of the three-dimensional mapping system Carto, the Pentaray mapping electrode was used to perform three-dimensional reconstruction and activation mapping of the right atrium (Figure 2A). According to the results of the Carto mapping, it was confirmed that the atrial tachycardia lesion was located at the apex of the right atrial ear. In this area, the surgeon used the Navi Star Thermo-cool SMART TOUCH ablation catheter (Johnson & Johnson Family of Companies, USA) to meticulously map the earliest activation point— the A wave of the bipolar electrogram was ahead of the body surface atrial wave 44 ms, and the unipolar electrogram showed a QS shape (Figure 2B). The ablation was performed, with setting the power as 15–25 W, the temperature as 43°C, the saline perfusion rate as 17 ml/min, and the ablation time as 30–40 s at each ablation point. 50 radiofrequency injuries were performed in a total of 50 min, but failed to terminate AT. At the end of the operation, no pericardial effusion was found on transthoracic echocardiography.

**Figure 3:**
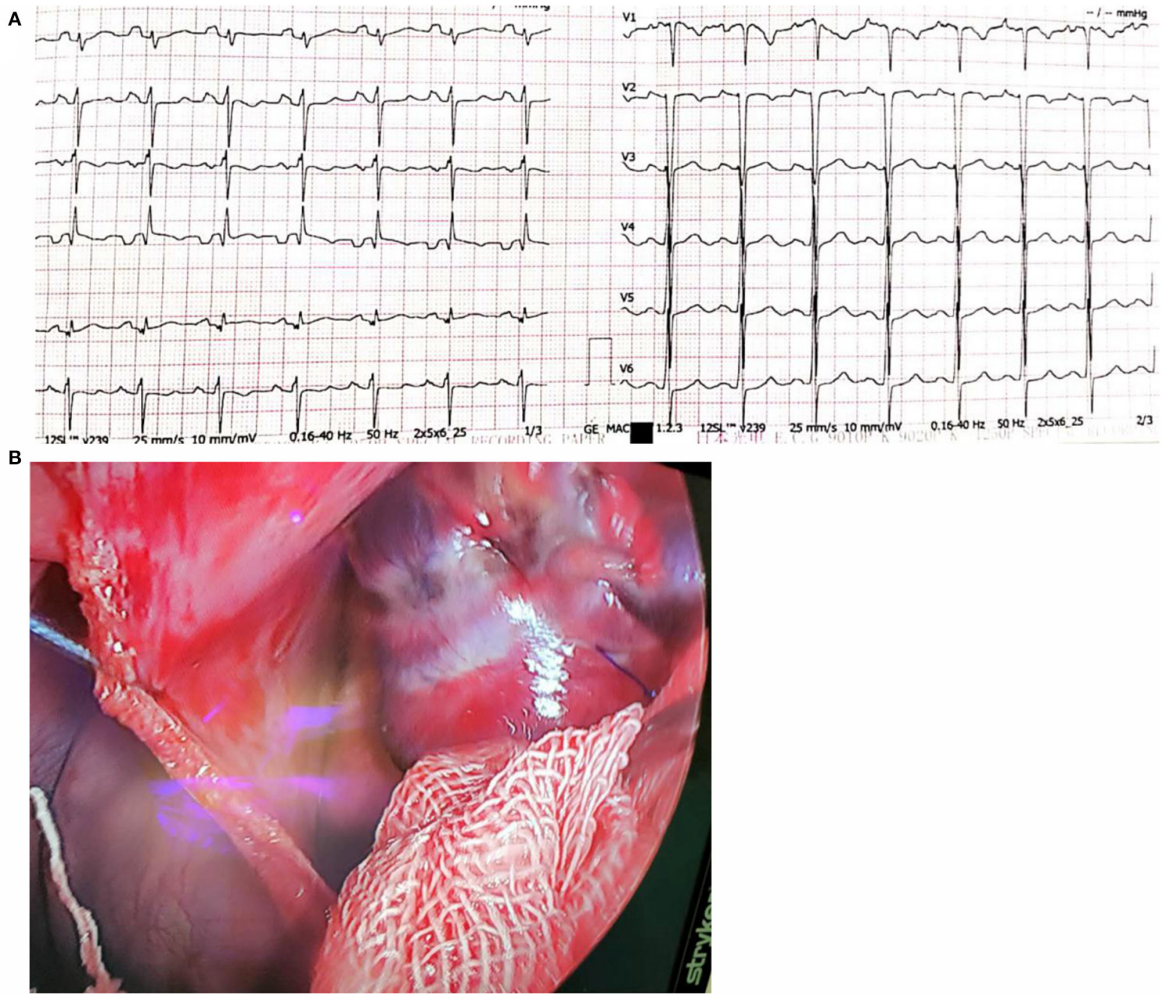
**(A)** The surface electrocardiogram showing sinus rhythm after surgical ablation; **(B)** The adventitia scar after ablation of the right atrial appendage under thoracoscopic clamping.

Considering that endocardial radiofrequency ablation was not effective, the patient was transferred to the cardiology surgery department for thoracoscopic epicardial radiofrequency ablation. When entering the operating room, the patient presented persistent atrial tachycardia, with a ventricular rate of 190 beats per minute. Esophageal ultrasound showed that the patient's heart was enlarged, especially the left atrium and left ventricle. The left heart function was extremely diminished. The intraoperative EF value was about 20%. The patient was intubated under general anesthesia, and CVC and MAP tubes were placed. Patients underwent routine ECG monitoring and blood oxygen saturation testing during the operation. The supine position was taken for surgery. The surgeon routinely sterilized the drape, entered the chest through a 4 cm anterior axillary incision in the 4th intercostal space, and entered the thoracoscopy through a 1.5 cm mid-axillary incision in the 4th intercostal space. Next, the surgeon cut the pericardium longitudinally and hung it to fully expose the right atrial appendage. Finally, the surgeon used a bipolar radiofrequency ablation forceps (Medtronic, Inc., USA) to clamp the root of the right atrial appendage and perform radiofrequency ablation. Ablation was repeated for three times, with the power as 28.5 W, and the duration of each ablation was adjusted by the tissue conductance profile. After the operation, the patient's atrial tachycardia was terminated and returned to sinus rhythm, and the heart rate immediately went from 160 to 85 beats/min (Figure 3).

**Figure 4:**
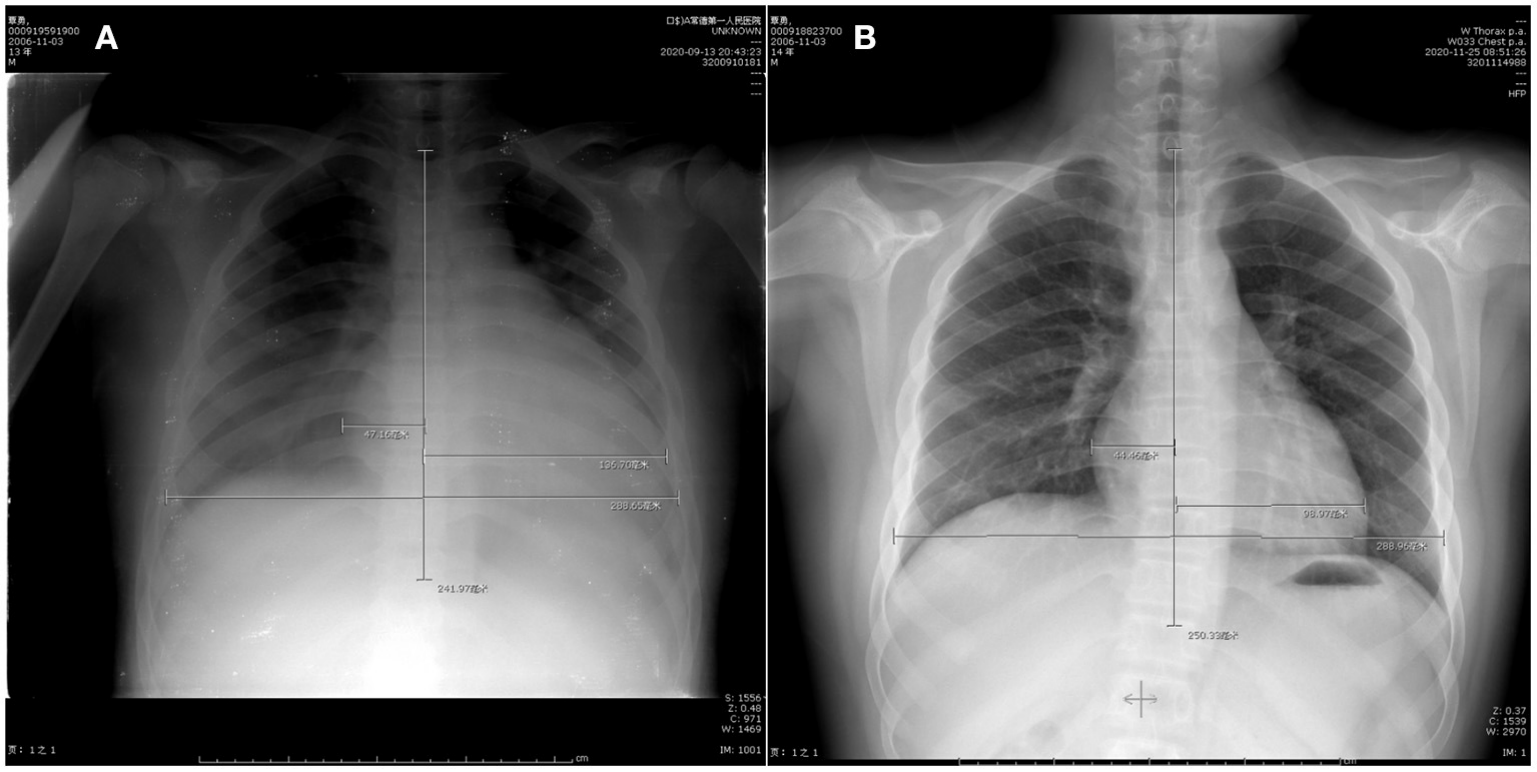
Chest radiographa **(A)** before operation, cardio-thoracic ratio 0.64; **(B)** 2 months after operation, cardio-thoracic ratio 0.50.

After the operation, the patients took sacubitril and valsartan, spironolactone, metoprolol sustained-release tablets, and potassium magnesium aspartate. The patient did not feel palpitations and no arrhythmia was detected by holter during the 3-month follow-up period after discharge from the hospital, and the heart structure gradually returned to normal. The chest radiograph showed that the cardiothoracic shrinkage to normal compared with the preoperative (Figure 4). The echocardiogram showed the diameter of left atrium and ventricle were 35 and 58 mm, respectively, and the left ventricular ejection fraction was 60%.

## Discussion

Patients with atrial tachycardia originating from the right atrial appendage are mostly young, with a longer course, and are more prone to persistent or endless seizures, which easily leads to tachycardia cardiomyopathy, poor drug efficacy, and risk of worsening arrhythmia. In this case, the heart of the child was enlarged with reduced ejection fraction, and heart failure occurred, which was in line with the clinical features of permanent atrial tachycardia that easily leaded to tachycardia cardiomyopathy (8). After successfully curing the atrial tachycardia, the heart structure returned to normal, suggesting that the cardiomyopathy caused by the tachycardia is reversible.

The right atrial appendage derives from the original atrium during the embryonic period, and its outer side moves with the free wall of the atrium body through the pectinate muscle. Because the right atrial appendage has the characteristics of free, thin wall, and large anatomical variability, excessive catheter operation tension or high radio frequency energy may cause right atrial appendage perforation. In addition, due to the lack of contractile force of the right atrial appendage, once the perforation is difficult to close on its own, pericardial tamponade is prone to occur, and surgical thoracotomy is often required for repair. Therefore, the right atrial tachycardia has the characteristics of easy mapping and difficult ablation. Part of the origin of atrial tachycardia is located at the apex of the right atrial appendage or the epicardium. It is more difficult for the ablation catheter to reach the accurate target, or the ablation energy cannot penetrate the wall. This will result in a higher chance of recurrence even if the intraoperative transient ablation is successful. Clinically, although there are some successful cases of endocardial radiofrequency ablation of right atrial tachycardia (9), most of them originate from the base of the right atrial appendage (10, 11). In this case, electrical activation mapping confirmed that the atrial tachycardia originated from the apex of the right atrial appendage, therefore, repeated attempts of endocardial radiofrequency ablation were unsuccessful.

For part of the catheter radiofrequency ablation failure, or the difficult and risky right atrial appendage leading atrial tachycardia, right atrial appendage resection may have a higher and longer lasting effectiveness. Especially for the atrial tachycardia originated from the apex of the right atrial appendage, surgical resection of the right atrial appendage may be more reasonable, more thorough, and have a better long-term prognosis (12). However, right atrial appendage resection has some unavoidable trauma, even a small parasternal incision still affects the appearance. Radiofrequency ablation of the right atrial appendage with bipolar ablation forceps under thoracoscopy can also obtain definite clinical curative effects, with less trauma and acceptable safety, and retains the structure and function of the right atrial appendage. Therefore, in this case, after the catheter ablation failed, we chose this surgical method because it has a good risk-benefit ratio. Of course, the success of the surgical operation must also have the premise of accurately judging the origin of the atrial tachycardia by the three-dimensional electrical activation mapping of the cardiology department.

In summary, when transendocardial ablation of atrial tachycardia originated from the right atrial appendage in young patients is not effective, thoracoscopic clamp radiofrequency ablation may be a reasonable choice.

## Data Availability Statement

The original contributions presented in the study are included in the article/supplementary material, further inquiries can be directed to the corresponding author/s.

## Ethics Statement

Written informed consent was obtained from the individual(s), and minor(s)' legal guardian/next of kin, for the publication of any potentially identifiable images or data included in this article.

## Author Contributions

RS and LG provide the concept of the manuscript. LL wrote the manuscript. ZQ revised the manuscript. All authors cared for the patient and approved the final manuscript.

## Conflict of Interest

The authors declare that the research was conducted in the absence of any commercial or financial relationships that could be construed as a potential conflict of interest.
