# Supplementary material for: Case Report: Application of Thoracoscopic Clamp Radiofrequency Ablation on Atrial Tachycardia Originating From Right Atrial Appendage After Catheter Ablation Failure
Source: Front Cardiovasc Med. 2021 Apr 28;8:659821. doi: 10.3389/fcvm.2021.659821 (PMC8113401; doi:10.3389/fcvm.2021.659821)
Supplement: Supplementary file 1 [file Data_Sheet_1.PDF]

CARE case report guidelines

CARE Checklist (2013) of information to include when writing a case report

(cc) BY-NC-ND

✔

Topic	Item	Checklist item description	Reported on Line
**Title**	**1**	The words "case report" should be in the title along with the area of focus	1
**Key Words**	**2**	2 to 5 key words that identify areas covered in this case report.	26
**Abstract**	**3a**	Introduction—What is unique about this case? What does it add to the medical literature?	13
	**3b**	The main symptoms of the patient and the important clinical findings	15
	**3c**	The main diagnoses, therapeutics interventions, and outcomes	16
	**3d**	Conclusion—What are the main "take-away" lessons from this case?	22
**Introduction**	**4**	One or two paragraphs summarizing why this case is unique with references	30
**Patient Information**	**5a**	De-identified patient specific information	41
	**5b**	Main concerns and symptoms of the patient	42
	**5c**	Medical, family, and psychosocial history including relevant genetic information (also see timeline).	53
	**5d**	Relevant past interventions and their outcomes	40
**Clinical Findings**	**6**	Describe the relevant physical examination (PE) and other significant clinical findings.	42
**Timeline**	**7**	Important information from the patient's history organized as a timeline	43
**Diagnostic Assessment**	**8a**	Diagnostic methods (such as PE, laboratory testing, imaging, surveys).	46
	**8b**	Diagnostic challenges (such as access, financial, or cultural)	50
	**8c**	Diagnostic reasoning including other diagnoses considered	51
	**8d**	Prognostic characteristics (such as staging in oncology) where applicable	71
**Therapeutic Intervention**	**9a**	Types of intervention (such as pharmacologic, surgical, preventive, self-care)	74
	**9b**	Administration of intervention (such as dosage, strength, duration)	76
	**9c**	Changes in intervention (with rationale)	79
**Follow-up and Outcomes**	**10a**	Clinician and patient-assessed outcomes (when appropriate)	91
	**10b**	Important follow-up diagnostic and other test results	91
	**10c**	Intervention adherence and tolerability (How was this assessed?)	88
	**10d**	Adverse and unanticipated events	NA
**Discussion**	**11a**	Discussion of the strengths and limitations in your approach to this case	126
	**11b**	Discussion of the relevant medical literature.	116
	**11c**	The rationale for conclusions (including assessment of possible causes)	140
	**11d**	The primary "take-away" lessons of this case report	141
**Patient Perspective**	**12**	When appropriate the patient should share their perspective on the treatments they received	124
**Informed Consent**	**13**	Did the patient give informed consent? Please provide if requested	**Yes** ☑ **No** ☐
